# Mating Delay Reduces Reproductive Performance but not Longevity in a Monandrous Moth

**DOI:** 10.1093/jisesa/ieaa009

**Published:** 2020-03-02

**Authors:** Xia-Lin Zheng, Junyan Liu, Wen Lu, Xiong Zhao He, Qiao Wang

**Affiliations:** 1 Guangxi Key Laboratory of Agric-Environment and Agric-Products Safety, National Demonstration Center for Experimental Plant Science Education, College of Agriculture, Guangxi University, Nanning, China; 2 School of Agriculture and Environment, Massey University, Palmerston North, New Zealand

**Keywords:** delayed mating, mating success, reproductive output, mating disruption

## Abstract

Age at mating is one of the most important factors that affect mating success and reproductive fitness in insects. The present study investigated how the age of the two sexes at mating determined mating success, reproductive fitness and longevity in *Phauda flammans* (Walker) (Lepidoptera: Phaudidae), a serious pest of *Ficus* spp. trees in South and Southeast Asia. The study may provide basic knowledge for the development of mating disruption programs using sex pheromones to control this pest. The species is monandrous and its adults live for only 4–5 d. We show that delayed mating significantly lowered mating success in both sexes, with males being more severely affected than females. Mating delay also reduced reproductive outputs of both sexes but females were more negatively affected than males. We did not find any effect of delayed mating on longevity of either sex. Our findings suggest that mating disruption with sex pheromones can be an effective method to delay mating in *P*. *flammans*, reducing reproductive success and thus limit population growth.

Age is an important factor that can affect mating success in insects (e.g., Amoah et al. 2019, Lai et al. 2020). Depending on the status of mates in an arena, either sex can be choosy. For example, males should be choosy when they contribute considerable investment to the offspring (Bonduriansky 2001, Edward and Chapman 2011, Ng et al. 2017, Kaufmann and Otti 2018) while in many cases males compete for access to females and females tend to be choosy (Kokko et al. 2003, Puurtinen and Fromhage 2017, Kelly 2018, Li and Holman 2018).

Age at mating is one of the most important elements influencing mate preference and fitness outcome in insects (e.g., Jiménez-Pérez and Wang 2003, Xu and Wang 2009, Martin et al. 2016, Kelly 2018, Lai et al. 2020). However, age-dependent mating success and fitness consequences appear to be divergent in different species. For example, in some species, females may prefer older males for mating to gain higher genetic quality for their offspring (Simmons 1995, De Luca 2015). In other species, females are more likely to mate with younger males to benefit from the higher breeding value and fewer deleterious mutations (Beck and Powell 2000, Beck and Promislow 2007). Previous studies show that males may prefer younger females to produce more progeny (Polak et al. 1998, Nandy et al. 2012, Mphosi 2019) or older females to gain greater fecundity and fertility (Wang et al. 2016, Lentini et al. 2018).

Recent studies demonstrate that delayed mating may increase adult longevity (Mori and Evenden 2013, Yang et al. 2017, Wu et al. 2018). Various authors suggest that in response to delayed mating, adults invest more in somatic maintenance for future reproduction (Proshold et al. 1982, Fadamiro and Baker 1999, Burke and Bonduriansky 2018) or mating opportunities (Milonoff 1995, Wu et al. 2018). However, mating delay can also reduce adult longevity (Leather et al. 1985, Bakker et al. 2011, Ricciardi et al. 2019). For example, females experiencing delayed mating could allocate their resources to eggs immediately after fertilization, reducing their overall longevity (Bakker et al. 2011, De Loof 2011).

*Phauda flammans* (Walker) (Lepidoptera: Phaudidae) is a serious pest of *Ficus* spp. trees in China (Liu et al. 2016), India (Nageshchandra et al. 1972, Verma and Dogra 1982), Vietnam (Fof 2015), and Thailand (Anonymous 2018). Its biology (Liu et al. 2014, 2015a, 2015b, 2016; Zheng et al. 2017) and management (Zheng et al. 2015, 2019; Huang et al. 2019) are well investigated, making it a suitable model for the study of age-dependent mating success and fitness consequences. *Phauda flammans* is a monandrous moth and its adults live for 4–5 d (Liu 2018). Most moths could mate within the first or second day after emergence with mating peak between the ninth and tenth hours of the photophase (Zheng et al. 2019). So far, nothing is known about how the age of the two sexes at mating affects mating success, reproductive fitness and longevity in this species, knowledge of which will be of significance for the understanding of its mating system and development of its pest management programs such as mating disruption. For example, mating delays caused by mating disruption may reduce mating success, reproductive output and thus plant damage.

Based on the information outlined above, we postulate that in *P*. *flammans* 1) younger males and females achieve higher mating success; 2) mating delay of either sex reduces reproductive output; and 3) delayed mating increases longevity of both sexes. To test these hypotheses, we carried out mating trials with nine age combinations of the two sexes and recorded mating success, the number of eggs laid and fertilized, and adult longevity.

## Materials and Methods

### Insects

Mature *P*. *flammans* larvae were collected on *Ficus concinna* (Miq.) Miq. and *F*. *benjamina* L. at Guangxi University (108°29′E, 22°85′N), Nanning, China, in late June 2016. We reared them in 50 transparent plastic containers (15 × 10 × 8 cm), each with 20–30 pinholes (≈1 mm in diameter). We kept 30 larvae in each container with fresh *F*. *concinna* leaves as food. The leaves were replaced with fresh ones and the number of larvae counted once a day until pupation. Dead larvae, if any, were replaced with live ones. The pupae were sexed according to Mao et al. (2017) and kept individually in plastic tubes (2.7 × 10.3 cm) with a plastic lid bearing 10–15 pinholes (≈1 mm in diameter) to obtain virgin adults of different ages. Adults were given 10% sucrose solution (Liu et al. 2015b). The insects were maintained and all experiments carried out at 26 ± 2°C, 70 ± 10% relative humidity, and a photoperiod of 16: 8 (L:D) h.

### Effect of Adult Age on Mating Success

Because adult moths normally live for about 4 d (Liu 2018), we set up nine combinations of pairing (treatments): 3 female ages (1, 2, and 3 d old) × 3 male ages (1, 2 and 3 d old). Virgin moths of different ages were randomly selected and paired individually in a gauze cage (50 cm length × 50 cm width × 180 cm height) until death. Thirty replicates were performed for each treatment. Observation on each treatment started immediately after moths were paired using four digital video cameras (DS-2CD3T45D-13, Hikvision, Co., Ltd., Hangzhou, China). Pairs that mated successfully in each treatment were recorded to calculate the mating success rate (number of mated pairs / total number of observed pairs × 100%).

### Effect of Moth Age at Mating on Reproductive Performance and Longevity

We provided fresh *F*. *concinna* branches with leaves in each of the above 30 cages as oviposition substrates, which were replaced daily. The number of eggs laid on the leaves of branches by each mated female was counted daily under a stereomicroscope (M205C, Leica, Germany). All eggs (<24 h old) laid by each mated female were introduced into a transparent plastic Petri dish (10 cm in diameter) for hatching. They were checked three times (0600, 1400, and 2200 hours) a day and hatch rate (the number of hatched larvae / total number of eggs laid × 100%) was recorded. The longevity of both sexes was recorded.

### Statistical Analysis

Data analysis was performed using SAS software (SAS Institute 2011). Normality of data was tested prior to analysis using a Shapiro–Wilk test (UNIVARIATE Procedure). Data on mating success, fecundity and egg hatch rate affected by female age and male age were normally distributed and thus analyzed using a linear regression model (GLM Procedure): mating success (%), fecundity or egg hatch (%) = *a × *male age + *b × *female age + *c × *male age × female age, where *a*, *b* and *c* are the regression coefficients (i.e., slopes). Only significant parameters were included in the final model. Data on adult longevity were not normally distributed even after transformation, and thus analyzed using a nonparametric analysis of variance (ANOVA; GLM Procedure) followed by Bonferroni (Dunn) *t* Tests for multiple comparisons. A level of *P* < 0.05 was accepted as statistically significant.

## Results

### Effect of Adult Age on Mating Success

**Fig. 1.**
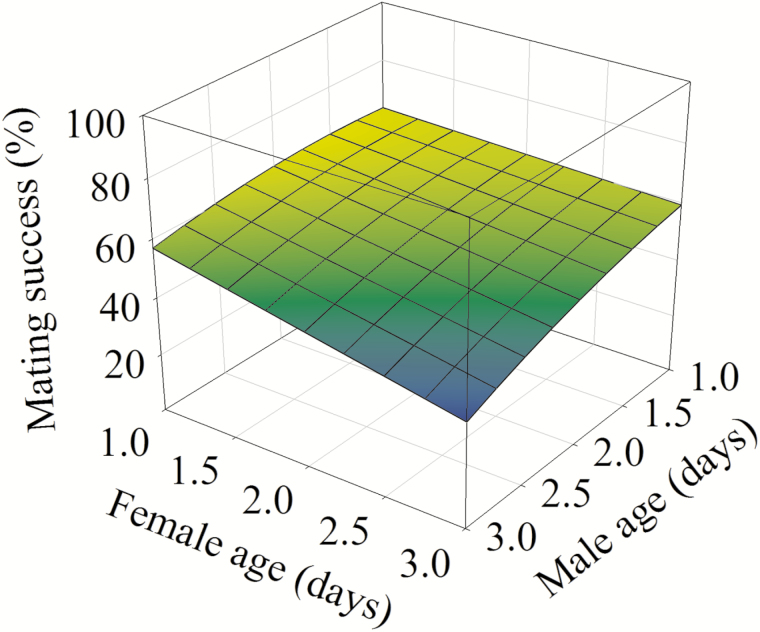
Effect of female and male age on mating success in *P*. *ﬂammans.* Mating success (%) = 60.00 + 10.31 × male age—2.78 × male age^2^ – 3.21 × male age × female age (*F* = 146.68; df = 3, 5; *P* < 0.001; *R*^2^ = 0.989).

Mating success significantly decreased with the increase of male age (*F* = 10.99, df = 1, 5, *P* = 0.021) (Fig. 1). Although female age alone did not affect mating success significantly (*F* = 6.12, df = 1, 4, *P* = 0.069), male and female age had significant interactions on mating success and with the increase of age in both sexes, mating success significantly decreased (*F* = 206.13; df = 1, 5; *P* < 0.001) (Fig. 1).

### Effect of Adult Age at Mating on Reproductive Fitness and Longevity

**Fig. 2.**
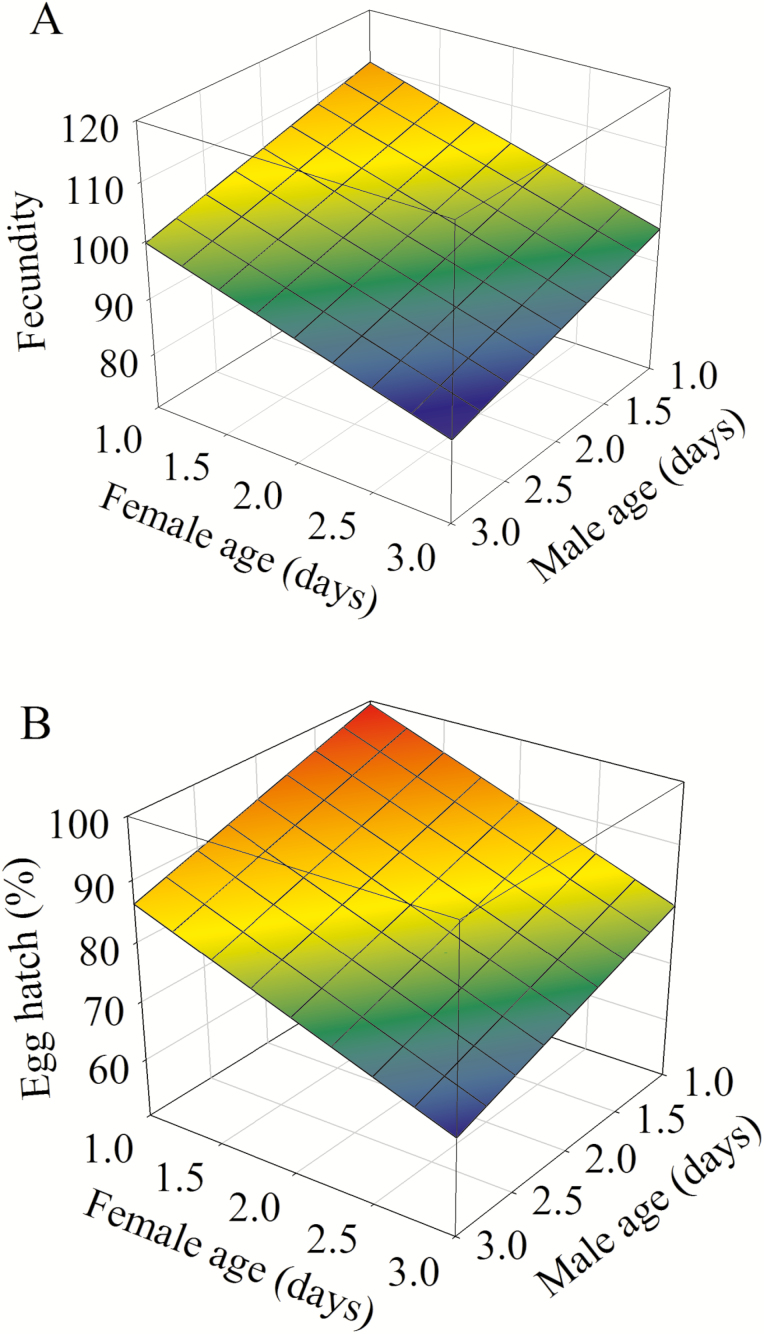
Effects of female and male age on fecundity and egg hatch (%) in *P*. *ﬂammans*. (A) fecundity = 123.81 – 5.54 × male age—7.48 × female age (*F* = 15.79; df = 2, 145; *P* < 0.001; *R*^2^ = 0.179); (B) egg hatch (%) = 97.32 – 2.62 × male age—3.63 × female age (*F* = 12.78; df = 2, 145; *P* < 0.001; *R*^2^ = 0.150).

Age of both sexes at mating significantly negatively affected fecundity (*F* = 21.77, df = 1, 145, *P* < 0.001 for female; *F* = 11.66 *df* = 1, 145; *P* < 0.001 for male; Fig. 2A) and egg hatch rate (*F* = 17.91, df = 1, 145; *P* < 0.001 for female; *F* = 9.12, df = 1, 145, *P* = 0.003 for male; Fig. 2B). However, adult age at mating had no effect on female (mean ± SE, 4.82 ± 0.29 d; range, 4.00–5.26 d) and male longevity (mean ± SE, 3.66 ± 0.21 d; range, 3.41–4.00 d) (*F* = 1.94, df = 8, 139, *P* = 0.059 for female; *F* = 0.62, df = 8, 139, *P* = 0.758 for male).

## Discussion

The present study demonstrates that the age at mating negatively affected reproductive performance in the monandrous moth *P*. *ﬂammans* but the extent of the impact differed between the sexes. We reveal that aging in males or both sexes reduced mating success significantly (Fig. 1). This suggests that senescence has more negative influence on male mating success and females are choosier in terms of mates’ age in this species, probably because eggs are more expensive than sperm (Darwin 1871). Because younger males carry fewer deleterious mutations (Beck and Powell 2000, Beck and Promislow 2007), females could also benefit from mating with young mates. Similarly, aging affects males more severely than females in mating success in a moth *Cnephasia jactatana* Walker (Jiménez-Pérez and Wang 2003), and females prefer to mate with younger males, resulting in lower mating success in older males in another moth *Dendrolimus punctatus* Walker (Lai et al. 2020).

Previous studies indicate that mating delay in both sexes results in significantly lower mating success in *Cnaphalocrocis medinalis* Guenée (Kawazu et al. 2014) and *Spodoptera litura* (Fabricius) (Wu et al. 2018). However, similar to *Lobesia botrana* (Denis and Schiffermüller) (Torres-Vila et al. 2002) and *Planococcus ficus* (Signoret) (Lentini et al. 2018), female age alone did not affect mating success significantly in *P*. *ﬂammans* (Fig. 1). This may be because males are less choosy, tending to mate as many females as possible (Bateman 1948), or older females reduce choosiness and require less courtship (Moore and Moore 2001). With variable age of both males and females, we indicate that the age of the sexes had significant interactions in mating success and when both sexes were oldest, their mating success reached the minimum (Fig. 1), suggesting that female age also plays a role in mating success when males are older.

We show that the age at mating of both sexes significantly reduced fecundity and fertility in *P*. *ﬂammans* (Fig. 2), corroborating previous reports on several other insect species (e.g., Kawazu et al. 2014, Yang et al. 2017, Dhillon et al. 2019). Our data do not support the notion that older males are of higher quality (Simmons 1995, De Luca 2015) and older females lay more eggs on average (Wang et al. 2016, Lentini et al. 2018). Similar to *S*. *litura* (Wu et al. 2018) and *Lasioderma serricorne* (F.) (Amoah et al. 2019), the negative impact of aging on reproductive output of *P*. *ﬂammans* was more severe in females than in males (Fig. 2), suggesting that eggs are more sensitive than sperm to senescence of their carriers. So far, many studies have demonstrated that mating delay drastically reduces reproductive output of female moths (e.g., Torres-Vila et al. 2002, Mori and Evenden 2013). However, in a seed beetle *Acanthoscelides obtectus* (Say) (Maklakov et al. 2007), females that mate early in life suffer a significant reduction in lifetime fecundity probably because there are direct costs associated with mating early in life for females of this species.

The effect of mating delay on adult longevity varies in different species. In some species, delayed mating may increase the adult longevity (Mori and Evenden 2013, Yang et al. 2017, Wu et al. 2018) to reduce energy expenditure for future reproduction (Proshold et al. 1982, Fadamiro and Baker 1999, Burke and Bonduriansky 2018) or mating opportunities (Milonoff 1995, Wu et al. 2018). Mating delay can also reduce adult longevity in several other species (Leather et al. 1985, Bakker et al. 2011, Ricciardi et al. 2019) probably because delayed mating triggers females to quickly allocate their resources to eggs (Bakker et al. 2011, De Loof 2011) and males to ejaculate (Harwood et al. 2015) immediately after mating, resulting in early death. However, our data demonstrate that, similar to several species (e.g., Maklakov et al. 2007, Jones et al. 2008, Kawazu et al. 2014, Amoah et al. 2019), adult age at mating had no significant effect on female and male longevity in *P*. *ﬂammans*. It is thus likely that these species do not experience a trade-off between reproduction and longevity (Moore and Moore 2001). Furthermore, adults that live a very short life in monandrous species like *P*. *ﬂammans* may not be able to extend (or shorten) their longevity to trade-off for reproduction.

Overall, the present study provides the first empirical evidence for the effect of mating delay on reproductive fitness in the monandrous pest *P*. *ﬂammans*. We show that older males and females have lower mating and reproductive success. Adult age at mating neither increases nor reduces female and male longevity. Our findings suggest that mating disruption using female sex pheromones can be an effective method to delay mating in *P*. *flammans*, reducing reproductive success and thus suppressing population growth.
